# Research Progress on the Antibacterial Activity of Natural Flavonoids

**DOI:** 10.3390/antibiotics14040334

**Published:** 2025-03-22

**Authors:** Zhijin Zhang, Mingze Cao, Zixuan Shang, Jing Xu, Xu Chen, Zhen Zhu, Weiwei Wang, Xiaojuan Wei, Xuzheng Zhou, Yubin Bai, Jiyu Zhang

**Affiliations:** 1 Key Laboratory of New Animal Drug Project of Gansu Province, Lanzhou 730050, China; hnnydxzzj@163.com (Z.Z.); 13483458376@163.com (Z.S.); 82101221334@caas.cn (J.X.); chenxu000310@126.com (X.C.); wangweiwei@caas.cn (W.W.); weixiaojuan@caas.cn (X.W.); zhouxuzheng@caas.cn (X.Z.); 2 College of Life Science and Food Engineering, Hebei University of Engineering, Congtai District, Handan 056038, China; caomignze@126.com (M.C.); zhuzhen234@yeah.net (Z.Z.); 3 Key Laboratory of Veterinary Pharmaceutical Development of the Ministry of Agriculture and Rural Affairs, Lanzhou 730050, China; 4 Lanzhou Institute of Husbandry and Pharmaceutical Sciences of CAAS, Lanzhou 730050, China

**Keywords:** natural flavonoids, multidrug-resistant bacteria, antibacterial activity, antibacterial mechanism, synergistic effects

## Abstract

The use of antibiotics has greatly improved the treatment of bacterial infections; however, its abuse and misuse has led to a rapid rise in multidrug-resistant (MDR) bacteria. Therefore, the search for new antimicrobial strategies has become critical. Natural flavonoids, a class of widely existing phytochemicals, have gained significant research interest for their diverse biological activities and antibacterial effects on various drug-resistant bacteria. This review summarizes the latest research progress on flavonoids, with a particular focus on several flavonoids exhibiting certain antibacterial activity, and explores their antibacterial mechanisms, including disruption of cell membranes and cell walls, inhibition of proteins and nucleic acids, interference with signal transduction, suppression of efflux pump activity, and inhibition of biofilm formation and virulence factor production. Additionally, we have reviewed the synergistic combinations of flavonoids with antibiotics, such as the combination of quercetin with colistin or EGCG with tetracycline, which significantly enhance therapeutic efficacy.

## 1. Introduction

Antibiotics are the cornerstone of the modern healthcare system, revolutionizing the treatment of bacterial infections and saving millions of lives [1,2]. However, the abuse and misuse of antibiotics has led to the emergence of multidrug-resistant bacteria at an alarming rate, posing a serious threat to human and animal health. In 2019, nearly 5 million deaths worldwide were linked to antimicrobial resistance (AMR) [3], with 10 million deaths expected by 2050, more than the current number of deaths caused by cancer [4,5]. International organizations such as the World Health Organization have identified AMR as a major risk to public health [6]. Facing the challenges posed by drug resistance, it is essential to explore alternative drugs with potent antibacterial activity and unique mechanisms of action. A wide range of phytochemicals have been found to be potential antimicrobials, including terpenes, essential oils, alkaloids, lectins, peptides, and phenolic compounds [7,8]. Phenolic compounds encompass flavonoids (e.g., quercetin) [9], phenolic acids (e.g., caffeic acid) [10], tannins (e.g., tannic acid) [11], lignins (e.g., lignin) [12], coumarins (e.g., coumarin) [13], and more. Among these compounds, a considerable number of natural flavonoids have attracted the interest of many researchers due to their widespread presence, low toxicity, and high activity against drug-resistant bacteria [14,15].

**Figure 1:**
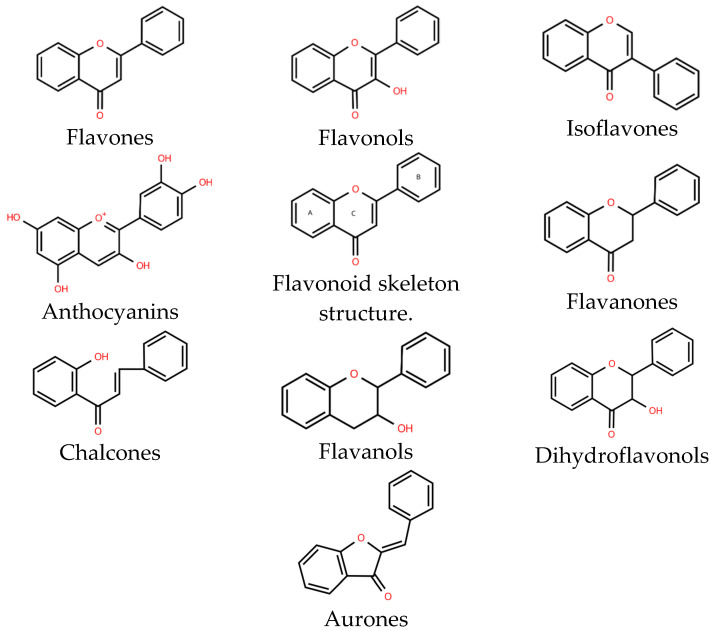
Chemical structure of nine major flavonoids.

Natural flavonoids are abundant phytochemicals widely present in various plants. Originally, flavonoids referred to a class of compounds derived from 2-phenylchromogen as the skeleton, and now generally refers to a series of compounds formed by two benzene rings connected by three carbon atoms, that is, a class of compounds with C_6_-C_3_-C_6_ structure (Figure 1) [16]. Based on the oxidation state and saturation of the heterocyclic ring, flavonoids can be mainly categorized into nine classes, including flavones (e.g., apigenin and luteolin), flavonols (e.g., quercetin and myricetin), flavanones (e.g., hesperidin and aringenin), flavanols or catechins (e.g., epicatechin and gallic catechins), chalcones (e.g., licochalcone A), dihydroflavonols (e.g., dihydromyricetin), aurones (e.g., aureusidin), anthocyanins (e.g., cyanidin), and isoflavones (e.g., genistein and daidzein) (Figure 1) [17]. Recently, flavonoids have been widely used in clinics for the control of different human diseases [18,19,20]. In addition to their known antioxidant, antidepressant, neuroprotective, anti-inflammatory, and anticancer activities, flavonoids have gradually received widespread attention due to their favorable antibacterial effects against resistant bacteria such as methicillin-resistant *Staphylococcus aureus* (*S. aureus*, MRSA) and amoxicillin-resistant *Escherichia coli* (*E. coli*, *AREC*) [21,22].

Significant progress has been made in research on the antibacterial activity of flavonoids. Existing literature reviews have summarized the antibacterial effects and mechanisms of flavonoids [23,24,25], but these studies are mostly limited to single mechanisms of action or specific types of flavonoids. This review primarily compiles relevant literature from 2014 to 2024, sourced from PubMed, Web of Science, Google Scholar, and Science Direct, using keywords such as “flavonoids”, “antibacterial and flavonoids”, “antibacterial mechanism and flavonoids”, “multidrug resistance”, and “synergistic effect of flavonoids”. It systematically summarizes the antibacterial activities, mechanisms of action, and synergistic effects of various flavonoids is intended to provide readers with a comprehensive understanding of these compounds.

## 2. Antibacterial Activity of Natural Flavonoids

Recently, many researchers have conducted comprehensive studies on the antibacterial activity of natural flavonoids, revealing various flavonoids with different degrees of antibacterial activity against bacteria (Table 1).

**Table 1:** The antibacterial effect of some flavonoids.

Category	S.NO.	Flavonoids	Chemical Structure	Microorganism	MIC (μg/mL)	Action Mechanism	References
Flavones	1	Apigenin	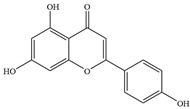	Quinolone-resistant *S. aureus* Mu50	4	Targeting the gyrA subunit with the Ser84Leu mutation	[26]
				Meticillin-susceptible *S. aureus* FDA 209P	>128	Targeting the gyrA subunit with the Ser84Leu mutation	[26]
				*P. aeruginosa* PAO1	64	-	[27]
				*Klebsiella pneumoniae* ATCC 9997	64	-	[27]
	2	Luteolin	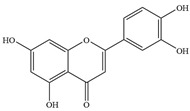	MDR *Trueperella pyogenes*	-	Downregulating the expression of MATE-encoded efflux pump	[28]
				Clinical isolation MRSA	500	Damage to cell wall and membrane integrity	[29]
				*P. aeruginosa* PAO1	64	-	[27]
				*Klebsiella pneumoniae* ATCC 9997	64	-	[27]
	3	Patuletin	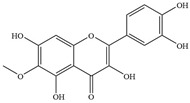	*S. aureus* ATCC 27853 and clinical strains	2000	Inhibiting biofilm formation and production of virulence factor glucanthin	[30]
	4	Chrysin	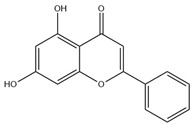	*S. aureus*	2–16	Inhibition of α-hemolysin expression	[31]
	5	Diosmetin	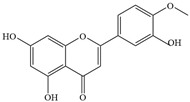	*S. aureus*	>128	-	[32]
	6	Tangeritin	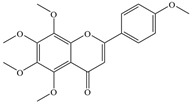	*E. coli*	137	Inhibition of DNA gyrase	[33]
	7	Nobiletin	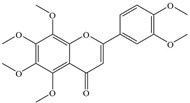	*E. coli*	177	Inhibition of DNA gyrase	[33]
	8	Acacetin	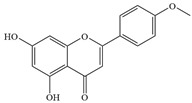	*Streptococcus pneumoniae (S. pneumoniae)*	>32	Inhibiting the formation of oligomers of pneumolysin (PLY) to attenuate its biological activity	[34]
	9	Wogonin	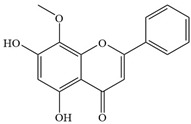	*S. aureus*	>128	-	[35]
	10	Scutellarein	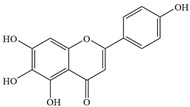	*A. baumannii*	1024	Inhibiting polyphosphate kinase 1 (PPK1)	[36]
	11	Entadanin	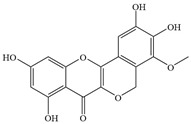	*S. typhi*	1.56	-	[37]
Flavonols	1	3′,4′, 7-trihydroxyflavone	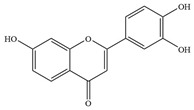	*E. coli* ATCC8739	32	Inhibiting efflux pump activity and enhancing the action of antibiotics	[38]
				*Klebsiella pneumoniae* ATCC 11296	32	Inhibiting efflux pump activity and enhancing the action of antibiotics	[38]
				*P.aeruginosa* PAO1	64	Inhibiting efflux pump activity and enhancing the action of antibiotics	[38]
	2	Rhamnetin 3-O--β-D-glucopyranoside	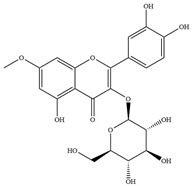	*S.aureus* USA300	-	Regulating carbon or glutamine/glutamate metabolism and reducing precursors of biofilm formation, thereby inhibiting biofilm formation	[39]
	3	Kaempferol	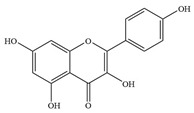	*S. aureus* ATCC 29213	>1024	Inhibiting the activity of sorting enzyme A to affect the formation of biofilm	[40]
				*S. mutans* ATCC 25175	1000	Inhibiting the enzyme activity of F-ATPase, destroying the ΔpH on the cell membrane, affecting the acidity of Streptococcus mutans	[41]
				*Pneumococcus* NCTC 7466	-	Decreasing the biological activity of sorting enzyme SrtA, inhibiting the formation of biofilm, and inhibiting the hemolytic activity of pulmonolysin	[42]
				*S. epidermidis* DMST 5038	>1024	-	[43]
	4	Kaempferol-3-O-α-l-rhamnoside	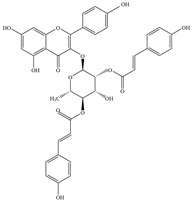	*S. aureus* 1199B	0.78	Inhibition of NorA transporter	[44]
	5	Quercetin	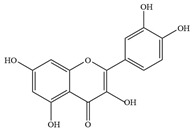	*S. epidermidis* DMST 5038	256	Damaging the cell membrane and increasing its permeability	[45]
				MRSA_A MRSE_178	-	Inhibiting the activity of ATP synthase and reducing the level of ATP in cells; virulence-inhibiting factor coagulase	[43,45]
				MRSA ATCC 33591	-	Stably binding to the formation site of SarA dimer, inhibiting biofilm formation, and reducing extracellular polymer production and eDNA concentration	[46]
				*P. aeruginosa* NCIM 20136	20	-	[47]
				*E. coli* NCIM 2065	400	-	[47]
				*Serratia marcescens* ATCC 14756	175	Inhibition of EPS production and biofilm formation	[48]
				*S. pyogenes* DMST 30653	128	Damaging the cell membrane and increasing its permeability	[49]
	6	Morin	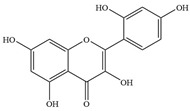	*S. pyogenes* MGAS 6180	-	Inhibition of biofilm formation	[50]
				*S. enteritidis* #1	150	Inhibition of DNA synthesis	[51]
				*Bacillus cereus* ATCC11778	300	Inhibition of DNA synthesis	[51]
	7	Myricetin	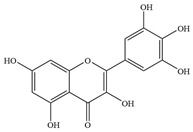	*E. coli* ATCC 25922	-	Inhibition of DnaB helicase	[52]
				*S. aureus* NCTC 8325-4	>1024	Inhibiting the production of virulence factor Hla and neutralizing Hla activity	[53]
				*S. aureus* ATCC 6538p	>1024	Inhibiting sorting enzymes A (SrtA) and B (SrtB) activity	[54]
	8	Fisetin	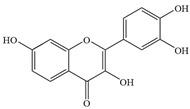	*NDM-1*-positive *E. coli*	>1024	Inhibiting New Delhi metallo-β-lactamase-1 (NDM-1) activity	[55]
	9	Galangin	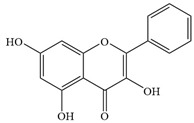	Penicillin-resistant *S. aureus*	200–300	Inhibiting the activity of penicillinase and β-lactamase	[56]
	10	Astragalin	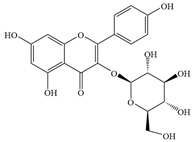	*H. pylori*	0.49–1.25	Interacts with proteins	[57]
	11	Rutin	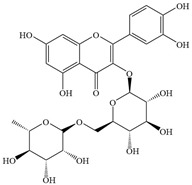	*P. aeruginosa*, MRSA	500–1000	Inhibiting biofilm formation, downregulating gene expression, destruction of cell membrane	[58]
Flavanones	1	Pinocembrin	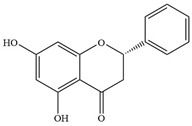	*Neisseria gonorrhoeae* GC1–182	64	-	[59]
				*Listeria monocytogenes* ATCC19113	68	-	[60]
	2	Hesperidin	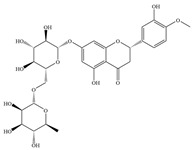	*E. coli* ATCC 25922	-	Inhibition of biofilm formation	[61]
				*S.aureus* ATCC 25923	512	-	[62]
				*P. aeruginosa* IBRS P001	500	-	[58]
	3	Hesperetin	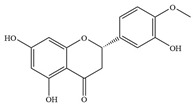	*Helicobacter pylori* ATCC 49503	30.23	Reducing the expression level of bacterial replication and transcription genes and inhibiting bacterial movement	[63]
				MRSA IBRS MRSA 011	500	-	[58]
				*P.aeruginosa* IBRS P001	500	-	[58]
	4	Naringin	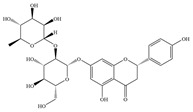	Clinical isolation of *Pseudomonas* species	128–512	Inhibition of EPS production and biofilm formation	[64]
				*S.aureus* ATCC 25923	512	-	[62]
				*P. aeruginosa* IBRS P001	250	-	[58]
				*E. coli* IBRS E003	500	-	[58]
	5	Naringenin	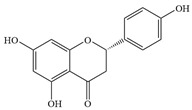	*S. mutans*	100–200	Inhibition of biofilm formation	[65]
	6	Eriodictyol	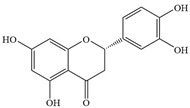	*S.aureus*	512	Inhibiting alpha-hemolysin expression	[66]
	7	Liquiritigenin	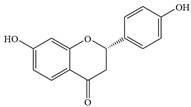	*S.aureus*	50–100	Inhibiting alpha-hemolysin expression and may inhibit β-lactamase activity	[67,68]
	8	7-Hydroxyflavanone	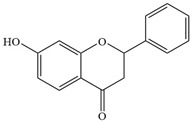	*S. pneumoniae*	1000	-	[69]
	9	Lupirifolin	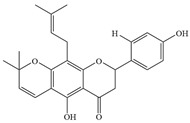	*S. aureus and Enterococcus faecalis (E. faecalis)*	0.5–2	Binds to phosphatidylglycerol (PG) and cardiolipin (CL) in bacterial cell membranes, thereby disrupting the integrity of the cell membrane	[70]
	10	Ochnaflavone	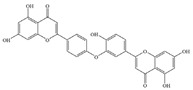	*P. aeruginosa*	31.3	-	[71]
	11	Sophoraflavanone G	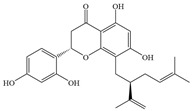	MRSA	3.9	Damages cell membrane, inhibits cell wall synthesis, interferes with energy metabolism	[72]
	12	Mimulone	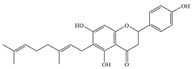	MRSA	2000–16,000	Inhibiting the synthesis of peptidoglycan layer in bacterial cell wall and inhibition of β-lactamase activity	[73]
	13	Sepicanin A	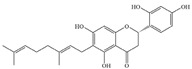	MRSA	998.7	-	[74]
Flavanols	1	Epigallocatechin gallate	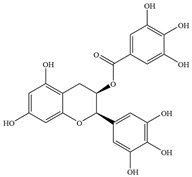	*S.aureus* ATCC 25923	100	Directly binding to peptidoglycan to interfere with the integrity of bacterial cell walls and biosynthesis and inhibiting penicillinase activity	[75]
				*Porphyromonas gingivalis* ATCC 33277	250	-	[76]
				*Vibrio parahaemolyticus* ATCC 17802	128	Damaging cell membrane integrity and inhibiting biofilm formation	[77]
	2	Epigallocatechin	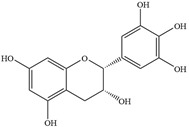	*Porphyromonas gingivalis* 381	1000	-	[76]
				*Porphyromonas gingivalis* ATCC 33277	1000	-	[76]
	3	Catechin	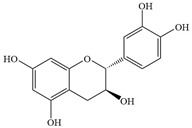	*E. coli*	1000–2000	Downregulating the acrA gene, thereby inhibiting biofilm formation	[78]
	4	(-)-Epicatechin	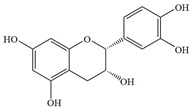	*S. Typhimurium*	>1024	Inhibition with ATP	[79]
	5	(-)-Epicatechin gallate	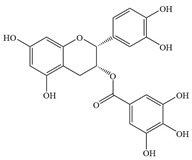	*S. Typhimurium*	>512	Inhibiting biofilm formation	[80]
	6	(-)-Catechin gallate	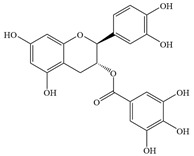	MRSA	256–512	Inhibiting biofilm formation and disrupting the secretion of virulence-related proteins	[81]
Isoflavones	1	Genistein	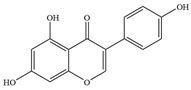	MRSA Newman 67-0	-	Inhibition of topoisomerase II and protein tyrosine kinase	[82]
				*E. coli* ATCC 25922	5	Inducing NO changes DNA and induces apoptosis	[83]
	2	Glabridin	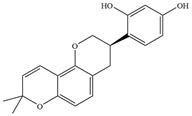	*MDRSA* 4627	-	Increasing oxidative stress, altering the integrity of DNA and proteins, and disrupting cell morphology	[84]
	3	Formononetin	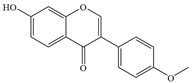	*S. aureus*	200	-	[85]
				*E. faecalis*	6.6–18.3	-	[86]
	4	Lupalbigenin	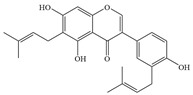	*S. aureus*, *E. faecalis*, and *Streptococcus pyogenes* (S. pyogenes)	4–8	Inhibiting α-hemolysin and biofilm formation, and damaging bacterial cell membranes	[87,88]
	5	Gancaonin M	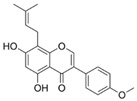	*S. aureus*, *E. faecalis*, *and S. pyogenes*	2–8	-	[87]
	6	Warangalone	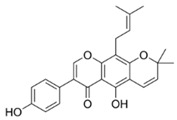	*S. aureus*, *E. faecalis*, *and S. pyogenes*	2–8	-	[87]
	7	Auriculatin	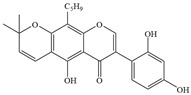	*S. aureus*, *E. faecalis*, *and S. pyogenes*	2–4	-	[87]
	8	Millexatin F	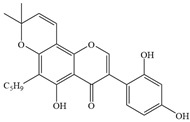	*S. aureus*, *E. faecalis*, *and S. pyogenes*	1–4	-	[87]
	9	Millexatin A	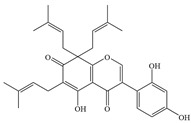	*S. aureus*, *S. epidermidis*, *and B. subtilis*	2–128	-	[89]
	10	Isolupalbigenin	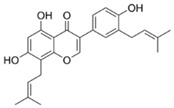	MRSA	1.56–3.13	-	[90]
	11	Erythrinin B	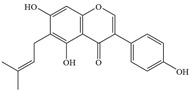	MRSA	6.25–12.5	-	[90]
	12	Laburnetin	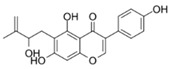	MRSA	>25	-	[90]
Anthocyanins	1	Cyanidin-3-*O*-glucoside	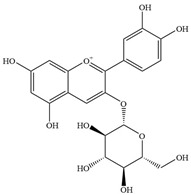	*S.aureus* ATCC 25923, SJS001, SJS008, SJS009, SJS010	312.5	Damaging cell membrane integrity	[84]
				*E. coli* ATCC 25922	5000	Damaging cell membrane integrity	[84]
	2	Delphinidin-3-glucoside	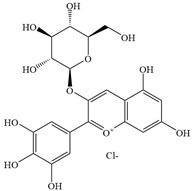	*E. coli*, *S.aureus*	1660–7110	-	[91]
	3	Delphinidin-3-sambubioside	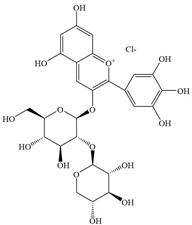	*E. coli*, *S.aureus*	1660–7110	-	[91]
	4	Pelargonidin-3-glucoside	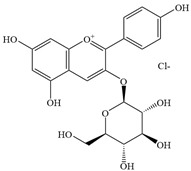	*E. coli*	-	Inhibition of microbial ATP synthase	[92]
	5	Malvidin-3-glucoside	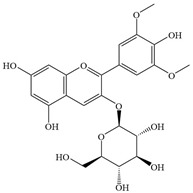	*Staphylococcus*	250	Inhibiting biofilm formation	[93]
	6	Cyanidin-3-rutinoside	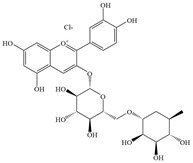	*P. aeruginosa*, *K. pneumoniae*	400–9500	-	[94]

### 2.1. The Mixture of Flavonoids Extracted from Plants

Studies on the antibacterial activity of flavonoids initially found that the mixed flavonoids extracted from plants had a significant inhibitory effect on microbial growth.

Zhong et al. [95] isolated six major flavonoids, including isoorientin, vitexin, isovitexin, rutin, quercetin, and kaempferol, from the crude methanol extract of Tartary buckwheat sprouts. The extract demonstrated significant inhibitory activity against *Salmonella typhimurium* (*S. typhimurium*) CMCC 50115, *Agrobacterium tumefaciens (A. tumefaciens)* ATCC 11158, *Pseudomonas lachrymans* (*P. lachrymans*) ATCC 11921, *Bacillus subtilis* (*B. subtilis*) ATCC 11562, *Staphylococcus albus* (*S. albus*) CICC 10897, and *Staphylococcus aureus* (*S. aureus*) ATCC 6538, indicating the antibacterial potential of these flavonoids with minimal inhibitory concentration (MIC) ranging from 800 to 3200 μg/mL. Lu et al. [96] extracted peony flavonoids from Fengdan peony seed meal, which demonstrated moderate antibacterial activity, with MICs of 29.3, 117.2, 234.4, and 7500 μg/mL against *S. aureus*, *Bacillus anthracis* (*B. anthracis*), *B. subtilis*, and *Bacillus perfringens* (*B. perfringens*), respectively. Wang et al. [97] extracted flavonoids from *Sedum aizoon* L. that were capable of inducing the disruption of the ultrastructural integrity of the fungus *Botrytis cinerea*, causing leakage of intracellular macromolecules such as nucleic acids and exerting antimicrobial effects by accumulating malondialdehyde and reactive oxygen species (ROS).

### 2.2. Single Flavonoids

To accurately assess their inhibitory effects on different bacterial strains, researchers began to focus on single flavonoids. This provides a scientific basis for developing new antibiotics, with a precise mechanism of action.

#### 2.2.1. Flavones

Apigenin and luteolin are flavones widely distributed in various plants, including sweet red pepper, parsley, chamomile, celery, and *Ginkgo biloba*, and have good antibacterial activity [98]. Yuh et al. [26] found that the MIC of apigenin against quinolone-resistant *S. aureus* Mu50 was 4 μg/mL, while the MIC against quinolone-susceptible *S. aureus* FDA 209P exceeded 128 μg/mL. Further research revealed that apigenin gained enhanced activity with the progression of quinolone resistance. Conversely, Mahamud et al. [99] found that luteolin could effectively inhibit the growth of *E. coli* and *S. typhimurium.* Luteolin enters the cells quickly by disrupting cell membrane integrity. Simultaneously, exogenous ROS enter the cell, causing oxidative damage, affecting the respiratory chain function and adenosine triphosphate (ATP) metabolism, and ultimately leading to bacterial death. Additionally, luteolin can completely inhibit pathogenic biofilm formation by preventing irreversible bacterial adhesion and reducing the cell surface hydrophobicity.

#### 2.2.2. Flavonols

Kaverol, rutin, baicalein, and quercetin are common flavonols found in saffron, scutellaria, lettuce, apples, and tea [98]. Research has revealed that quercetin exhibits good antibacterial effects against *E. coli*, *P. aeruginosa*, *S. typhimurium*, and *S. aureus*., demonstrating particularly higher sensitivity to Gram-positive bacteria, with MICs of 2.48, 2.57, 2.18, and 2.06 μg/mL, respectively. Treatment with quercetin has been found to cause cellular damage to both *E. coli* and *S. aureus*, leading to the disruption of their cell walls and membranes and ultimately resulting in leakage of intracellular enzymes, including β-galactosidase, alkaline phosphatase, and soluble proteins [100]. Moreover, quercetin has demonstrated good antibacterial activity against the plant pathogen *Xanthomonas axonopodis pv.* citri in vitro, with a half-maximal effective concentration (EC_50_) of 14.83 μg/mL, which is more effective than the organocopper broad-spectrum bactericide thiodiazole copper [101]. Baicalein, a flavonoid compound similar to quercetin, has also demonstrated certain antimicrobial activity. Wang et al. [102] found that baicalein can adhere to the cytoplasmic membrane phospholipids and the outer membrane lipopolysaccharides of Gram-negative bacteria (*Acinetobacter baumannii*), resulting in membrane rupture. Additionally, baicalein can increase ROS production within *A. baumannii*, inhibiting efflux pump activity and preventing bacterial biofilm formation, thereby enhancing doxycycline activity against *A. baumannii*. Baicalin, the glycoside form of baicalein composed of baicalein and glucuronic acid, exhibits certain antibacterial activity by destroying the cell wall and membrane integrity of *E. coli* (MIC for *E. coli* clinical isolates: 4000 μg/mL). Furthermore, baicalin has been found to enhance the susceptibility of *E. coli* isolates to antimicrobial agents such as streptomycin, ciprofloxacin, and ampicillin [103]. In addition to quercetin, baicalein, and baicalin, naringin, rutin, and kaempferol have certain antibacterial activity. Ivanov et al. [58] revealed that both naringin and rutin demonstrated certain antimicrobial activity against ten selected antibiotic-resistant bacterial strains. Notably, rutin has been found to reduce the ability of *P. aeruginosa* IBRS P001 to form biofilms. Li et al. [101] investigated the antimicrobial effects of 50 flavonoids on plant pathogenic bacteria and discovered that most of these compounds have moderate inhibitory effects on *Xanthomonas oryzae* and *Xanthomonas axonopodis pv.* citri. Specifically, kaempferol demonstrated moderate antimicrobial activity against *X. oryzae* in vitro, with an EC_50_ of 15.91 μg/mL.

#### 2.2.3. Flavanones

Naringin is a flavanone compound mainly found in citrus fruits [104]. Agus et al. [105] isolated naringin-rich parts from pigeon pea leaves and investigated their antibacterial effects against *S. typhi*, *S. aureus*, and *E. coli* through the paper diffusion method (Kirby–Bauer), revealing certain antibacterial activity against all tested bacteria, with an antibacterial zone diameter of 12.5, 11.0, and 9.5 mm, respectively. Yue et al. [65] found that the MIC of naringenin against *S. mutans* ranged from 100 and 200 μg/mL, with the growth curve demonstrating that both concentrations could significantly inhibit *S. mutans* growth. Additionally, 200 μg/mL naringin almost completely inhibited the biofilm formation by *S. mutans*. In addition, Zengin et al. [106] showed that naringenin had certain antibacterial activity against the clinically isolated strain *S. aureus* 105 with a MIC of 512 μg/mL. Weng et al. [72] found that sophoraflavanone G and matrone, two lavandulylated flavonoids in *Sophora flavences*, damaged the membrane integrity of *S. aureus* by targeting its bacterial membrane while inhibiting cell wall synthesis and preventing bacterial biofilm formation. These flavonoids also interfered with the energy metabolism of resistant *S. aureus*, hence demonstrating a bactericidal effect by impairing the normal physiological activities of bacteria. In addition to the aforementioned flavonoid compounds, pinocembrin and 7-*O*-methyleriodictyol also belong to the flavanones class and exhibit certain antibacterial activity against a variety of bacteria.

#### 2.2.4. Flavanols

Flavanols are 3-hydroxyl derivatives of flavones. They include the simplest monomers, catechins and epicatechins, as well as more complex structures like gallatechin, epigallocatechin, epigallocatechin gallate (EGCG), and proanthocyanidins, among others. These compounds are typically found in black and green teas and in fruits such as bananas, peaches, blueberries, and apples. They exhibit certain inhibitory effects against various bacteria [98]. Alkufeidy et al. [107] observed *S. aureus* treated with catechin extract from green tea by scanning electron microscopy and found that the bacterial cell membrane and cell wall morphological structure were damaged, resulting in cell disruption.

The EGCG isolated from green tea by Sakanaka et al. [76] completely inhibited the growth and adhesion of *Porphyromonas gingivalis* (*P. gingivalis*) on human buccal epithelial cells at a concentration of 250–500 μg/mL. Further studies revealed that this inhibitory effect was attributed to the presence of gallic groups in polyphenols linked to the 3-OH ester bond of catechins. Wang et al. [77] found that EGCG could not only damage the cell membrane of *Vibrio parahemolyticus* 17,802 (*V. parahemolyticus*), compromising its integrity but also inhibiting exopolysaccharide and biofilm formation in *V. parahemolyticus*. Noor et al. [108] found that a higher concentration of EGCG and theaflavin 3,3′-dialimate could inhibit the growth of *Clostridium perfringens*. In the presence of 250 μg/mL gallatechin gallate, the bacteria elongated without DNA separation and diaphragm formation. Zhang et al. [109] found that EGCG has MIC of 400 μg/mL for *Shigella flexneri* (*S. flexneri*), which can interfere with bacterial protein formation and change bacterial morphology. In addition, EGCG can perform an antibacterial role by reducing the level of superoxide dismutase (SOD) and increasing the level of ROS in bacteria.

#### 2.2.5. Anthocyanins

Cyanidin, pelargonidin, and peonidin are anthocyanins responsible for the coloration of fruits and blossoms, predominantly found in the outer cell layers of many fruits, including red grapes, raspberries, strawberries, blueberries, cranberries, and blackberries [98]. Li et al. [110] found that the MIC of cyanidin-3-*O*-glucoside (C_3_G) to both *S. aureus* and *E. coli* strains was less than 5000 μg/mL. Cyanidin-3-*O*-glucoside-lauric acid ester (C_3_G-LA) (MIC: 312.5 μg/mL) had stronger antibacterial activity against *S. aureus* strains than C3G, but the inhibitory effect on *E. coli* was the same as C_3_G. They effectively suppressed bacterial growth by destroying their cell membranes. Gong et al. [111] found that the MIC of cranberry anthocyanins against *S. aureus* was 5000 μg/mL, and treating bacteria with MIC of 2 for 0.5 h suppressed approximately 8 log CFU/mL of *S. aureus* due to decreased ATP and soluble protein levels, membrane structure damage, and cytoplasmic leakage. The anthocyanins extracted from pomegranate by Wafa et al. [112] had antibacterial effects on *Salmonella*, with MIC of 10,750–12,500 μg/mL. This concentration of anthocyanins significantly inhibited the growth of salmonella in chicken stored at 4 °C. Lacombe et al. [113] extracted anthocyanins from lowbush blueberries, with MIC of 34,750 μg/mL against *S. typhimurium* and a minimum bactericidal concentration (MBC) of 69,500 μg/mL. Compared to other categories of flavonoids, anthocyanins exhibit poorer antibacterial activity.

#### 2.2.6. Isoflavones

Genistein is an isoflavone which is mainly found in legumes such as soybeans [114]. Verdrengh et al. [82] found that the growth of *Streptococcus pasteurianus* (*S. pasteurianus*, *Bacillus cereus* (*B. cereus*)), and *S. aureus* strains LS-1, SKM 7 (srtB), SKM 12 (srtA-), SKM 14 (srtA-srtB-) and Newman was reduced 2- to 160-fold by the addition of 27.024 μg/mL genistein. This indicates that genistein has a good inhibitory effect on these bacteria. Formononetin is also an isoflavone compound with a good inhibitory effect against bacteria and fungi. Das Neves et al. [85] found that isoflavone formononetin extracted from red propolis had certain activity against all tested microorganisms, with MIC values of 200 μg/mL for six types of bacteria (*S. aureus* ATCC 13150, *S. aureus* ATCC 25923, *S. epidermides* ATCC 12228, *P. aeruginosa* ATCC 9027, *P. aeruginosa* ATCC P-12, and *P. aeruginosa* ATCC P-03). Hummelova et al. [115] found that biochanin A exhibits good antibacterial activity against *B. cereus*, *Listeria monocytogenes*, and *Streptococcus pyogenes*, with MICs ranging from 32 to 64 μg/mL. In addition to the aforementioned compounds, research has also found that several isoflavones, including orobol, gancaonin A, wighteone, millewanin H, furowanin A, senegalensin, and furowanin B7, exhibit certain antibacterial activity against the clinical strain *Streptococcus iniae* (*S. iniae*) DSJ19. Their MICs range from 7.81 to 500 µg/mL, while their MBCs range from 15.63 to 500 µg/mL [116].

The above studies have found that many flavonoids exhibit certain inhibitory effects against various bacteria and fungi, but their inhibitory effects and mechanisms against different pathogens vary significantly. Compared to traditional antibacterial agents, some flavonoids demonstrate stronger antibacterial activity, which provides a potential research direction for the development of novel antimicrobial drugs.

## 3. Antibacterial Mechanism of Flavonoids

**Figure 2:**
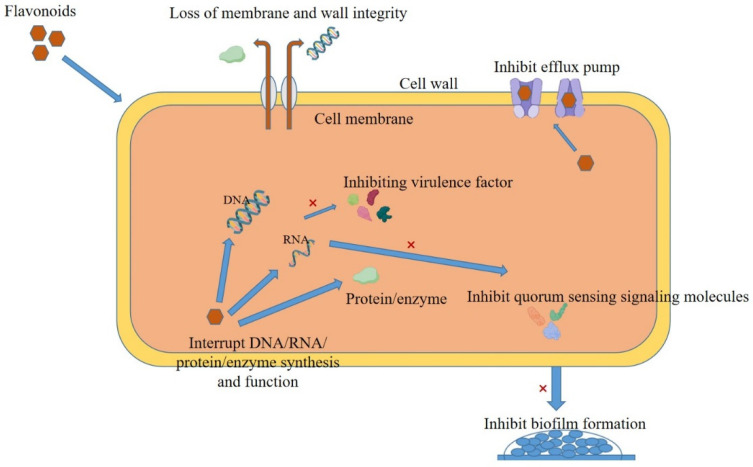
Antibacterial mechanism of natural flavonoids.

Flavonoids inhibit bacteria in various ways, including destroying their cell wall and membrane structures, affecting their normal morphology, inhibiting nucleic acid and protein synthesis or function, inhibiting biofilm formation, downregulating virulence factor expression, interfering with bacterial signal transduction, and inhibiting bacterial efflux pump (Figure 2).

### 3.1. Bacterial Cell Membrane Disruption

Cell membranes are the primary target sites of natural flavonoids against Gram-positive bacteria, including the disruption of the phospholipid bilayer and the inhibition of the respiratory chain by targeting quinone pools [62,117]. Studies have found that 128 μg/mL of quercetin can damage cell membrane integrity of amoxicillin-resistant *S. epidermidis*, leading to the leakage of bacterial DNA, RNA, and metabolites, thus affecting the normal growth of bacteria. Additionally, quercetin can enhance the inhibitory effect of amoxicillin on amoxicillin-resistant *S. epidermidis* by inhibiting β-lactamase activity [43]. Similarly to quercetin, kaempferol and EGCG can affect the normal growth of bacteria by disrupting cell membrane integrity. For instance, studies have found that kaempferol can kill *Helicobacter pylori* (*H. pylori*) by disrupting their cell membrane integrity. Kaempferol treatment increased the expression levels of *H. pylori* ABC transporters 1–4 and lolD 2 by a thousand-fold, indicating that the kaempferol-induced cell membrane damage in *H. pylori* may be related to these transporters [118]. Wang et al. [77] found that 256 μg/mL of EGCG could significantly disrupt cell membrane integrity in *V. parahemolyticus* 17,802, allowing propidium iodide to penetrate the bacteria and emit red fluorescence. Besides the above flavonoids, many other flavonoids can disrupt bacterial cell membrane integrity, resulting in leakage of internal proteins, nucleic acids, sodium ions, potassium ions, and other substances, ultimately resulting in bacterial death [119,120].

### 3.2. Bacterial Cell Wall Disruption

The cell wall is essential for bacterial growth, survival, and morphological maintenance. When the cell wall is disrupted, bacteria can easily rupture and die [121]. Liang et al. [122] found that dihydromyricetin is the most abundant flavonoid in vine tea extract, and *S. aureus* treatment with 1 MIC (6300 μg/mL and 1250 μg/mL, respectively) of vine tea extract and dihydromyricetin increased the extracellular alkaline phosphatase (AKP) concentration by 21.8% and 10.3%, respectively. Transmission electron microscopy revealed severe damage to the *S. aureus* cell wall, with blurred boundaries and bacteria autolysis after 12 h of treatment. Zhou et al. [123] found that flavonoids from *Chimonanthus salicifolius* S. Y. Hu. (with an MIC of 1560 μg/mL) exhibited a stronger inhibitory effect on Gram-positive bacteria than on Gram-negative bacteria. After 6 h of treatment, the extracellular AKP level in *S. aureus* was 6.97 times higher than that in the control group. Moreover, ultrastructural observations following bacterial treatment with flavonoids from salicylus flower revealed an irregular bacterial shape and cytoplasmic leakage, indicating disruption of cell wall integrity.

### 3.3. Impact on Bacterial Nucleic Acid

Nucleic acid plays a crucial role in bacterial growth and proliferation. Some flavonoids can interfere with the bacterial DNA/RNA replication and transcription process, causing changes in gene expression, and eventually, bacterial death. Kim et al. [63] found that hesperidin treatment decreased the mRNA expression levels of *H. pylori* replication-related genes (*dnaE*, *dnaN*, *dnaQ*, *holB*) and transcription-related genes (*rpoA*, *rpoB*, *rpoD*, and *rpoN*). The results indicated that hesperidin inhibited *H. pylori* growth by downregulating the replication and transcription of growth-related essential genes. Wang et al. [124] found that 28 h of treatment with soybean isoflavone reduced the fluorescence intensity of 4′,6-diaminidine 2-phenylindole by 60.18% in the *S. aureus* cell compared to the untreated control group, and the nucleic acid synthesis was significantly inhibited. Further studies revealed that soybean isoflavone inhibits the normal growth of bacteria by simultaneously acting on topoisomerases I and II to prevent nucleic acid synthesis. Morimoto et al. [26] found that apigenin targets the function of *gyrA* (a subunit encoding DNA gyrase) subunit with Ser84Leu mutation, inhibiting the quinolone-resistant *S. aureus* growth that carries a mutation in the quinolone resistance-determining region (QRDR) of the *gyrA* gene.

### 3.4. Proteins Affecting Bacteria

Some flavonoids can inhibit protein synthesis or bacterial function, such as inhibiting the activity of specific enzymes or interacting with target proteins, thereby blocking the protein synthesis and modification process, resulting in bacterial death. Griep et al. [52] found that myricetin inhibited the activities of *E. coli* DNA helicase and primers, and its sensitivity to DNA helicase was significantly higher than that of primers. Geethalakshmi et al. [125] found that flavonoids extracted from *Trianthema decandra* can interact with target proteins through four different amino acids, including glycine58 (GLY58), arginine98 (ARG98), selenomethionine56 (MSE56), and glutamic63 (GLU63). Their binding to *P. aeruginosa* dehydrase (FabZ) inhibits the enzyme’s activity, thereby affecting bacterial growth. *S. aureus* PriA is a helicase essential for restarting DNA replication and bacterial survival. Huang et al. [126] identified through the fluorescence quenching method that kaempferol could combine with PriA to form a complex and significantly inhibit PriA activity. Furthermore, certain flavonoids can inhibit enzyme activity by interfering with the binding of cofactors. Studies have shown that EGCG disrupts the cofactor NAD(P)H binding in both enzymes, effectively suppressing the FabG and FabI reductase steps in the bacterial fatty acid elongation cycle. This inhibition consequently blocks the bacterial type II fatty acid synthesis system, ultimately impairing bacterial growth [127].

### 3.5. Inhibition of Bacterial Biofilms

As a complex microbial assemblage, a biofilm is a group behavior of bacteria adapting to the environment to enhance their tolerance to the external environment and cause microbial infection, which can be difficult to eradicate. Biofilms pose a serious threat to human and animal health; therefore, inhibiting or eradicating them is crucial for inhibiting bacterial infection [128]. Wang et al. [129] found that the subinhibitory concentration of baicalin (250 μg/mL) downregulates the mRNA transcription levels of genes *srtA*, *uafA*, and *Aas* related to autolysis and surface protein production of azithromycin-resistant *S. saprophyticus* (*ARSS*), reducing *ARSS* surface protein production and eDNA release and causing bacterial autolysis. Additionally, baicalin can reduce the aggregation rate of bacteria in a dose-dependent manner, thereby inhibiting biofilm formation in *ARSS*, with a stronger inhibition effect in the adhesion and aggregation stages than in the mature stage of biofilm formation. Ivanov et al. [58] found that 500 and 250 μg/mL of rutin could significantly inhibit (100%) biofilm formation in urinary catheters of *P. aeruginosa* and MRSA. The removal rate of MBC rutin on *P. aeruginosa* and MRSA biofilms was 73.7% and 74.2%, respectively. Its inhibitory effect on biofilms was achieved by reducing cell viability, extracellular polysaccharide, and extracellular DNA levels. Qayyum et al. [130] found that quercetin could inhibit biofilm formation in *Enterococcus faecalis* (*E. faecalis*), with an inhibition rate proportional to the quercetin concentration; 64 μg/mL of quercetin could reduce the depth of the biofilm. Zhang et al. [109] found that 200 μg/mL of EGCG exhibited a 64.28% inhibition rate on the biofilm of *S. flexneri*. Its mechanism of action is to reduce the production of bacterial exopolysaccharides by inhibiting the *mdoH* gene expression, thereby affecting biofilm formation.

### 3.6. Inhibition of Bacteria-Related Virulence Factors

Bacterial virulence factors refer to the toxins, enzymes, and cell surface structures secreted by bacteria, such as lipopolysaccharides and lipoproteins, which can enhance their ability to evade host defense and cause diseases [131]. Xu et al. [42] found that kaempferol not only interferes with the pore-forming activity of PLY by binding to the catalytic active site, thereby inhibiting PLY-mediated cytotoxicity, but also significantly reduces the activity of sorting enzyme A by occupying its active site. Additionally, Yin et al. [132] found that kaempferol could inhibit α-hemolysin expression by downregulating *Hla* and *RNAIII* transcription, significantly inhibiting the hemolytic ability of *S. aureus*. Besides kaempferol, puerarin and rutin can inhibit the production of bacterial virulence factors. Tang et al. [133] found that puerarin exhibited a slight effect on the *S. aureus* growth; however, at a low dose of 8 μg/mL puerarin, the *Hla* expression was inhibited, and the production of bacterial α-hemolysin was significantly reduced. Ivanov et al. [58] found that 0.5 MIC of rutin could significantly reduce the production of extracellular virulence factors of *P. aeruginosa* IBRS P001. Compared with the control group, the production of elastase, anthocyanin, and rhamnolipid decreased by 91%, 82.1%, and 74.4%, respectively, and the production of protease decreased by >50%.

### 3.7. Effects on Bacterial Signaling Pathways

The bacterial signaling pathway is a series of molecular mechanisms in which bacteria respond to changes in the external environment. These mechanisms enable bacteria to quickly adapt to environmental changes to maintain their survival and reproduction ability. These signaling pathways mainly include quorum sensing (QS), different types of secretion systems (such as type I, II, III, and IV secretion systems), and metabolic signaling pathways.

Peng et al. [134] studied the effect of rutin on the QS system of avian pathogenic *E. coli* (*APEC*), revealing that rutin not only inhibited bacterial biofilm formation by reducing the secretion of signaling molecule autoinducer-2 (AI-2), but also significantly interfered with its QS by reducing the expression of *APEC* virulence gene. Lahiri et al. [135] found that catechins could significantly reduce the activity of QS protein LuxS of *P. gingivalis*. Nain et al. [136] found that quercetin and myricetin in the leaf extract of *Gynura procumbens* could interact with QS receptors LasR and RhlR of *P. aeruginosa*, thereby affecting bacterial QS. Zong et al. [137] found that baicalin dose-dependently downregulated the *luxS* expression in pork–enteral pathogenic *E. coli* and reduced the production of the QS signaling molecule AI-2, thus affecting the bacterial population dynamics. Chang et al. [138] found that chrysin not only competes with the natural signaling molecule C_6_HSL for the same binding site on the CviR receptor, but can also change its secondary structure, significantly preventing the binding of C_6_HSL/CviR and inhibiting bacterial QS. Zhang et al. [139] found that baicalin inhibits the type III secretion system (T_3_SS) through the Pseudomonas quinolone signal (PQS) system, significantly reducing the production of virulence factors in *P. aeruginosa* and weakening its toxicity. Lv et al. [140] found that myricetin not only inhibited the expression of Salmonella pathogenicity island 1 (*SPI-1*) effector proteins *sipA, sipB, and sipC*, but also significantly reduced the levels of *hilA*, *sopA*, *sicA*, and *prgH* proteins, which are primarily responsible for the secretion, regulation, and function of SPI-1-effector proteins. Further investigation revealed that the mRNA transcription levels of *hilD*, *hilC*, and *rtsA* and downstream genes in Salmonella were significantly reduced under induction conditions when myricetin was absent. This indicates that myricetin affects the transcription level of the *SPI-1* gene through the *hilD-hilC-rstA-hilA* regulatory pathway, thereby reducing the level of the key effector protein of *S. typhimurium* and inhibiting its T_3_SS-mediated virulence.

The above studies demonstrate that various flavonoids can affect bacterial signaling pathways, thereby affecting the normal growth of bacteria.

### 3.8. Inhibition of Bacterial Efflux Pump

Bacterial efflux pumps are complex systems that play crucial roles in bacterial physiology, metabolism, and pathogenicity. Inhibiting these pumps can significantly reduce bacterial resistance and restore their sensitivity to antibiotics. Lan et al. [141] found that five flavonoids extracted from *Artemisia rupestris* L. exhibited no inhibitory effect on MRSA when used alone (MIC > 128 μg/mL). However, when combined with antibiotics, they reduced the *NorA* expression at the mRNA level, thereby inhibiting the efflux pump in different MRSA strains. Zou et al. [142] found that biochanin A could act as an efflux pump inhibitor that not only inhibits the synthesis of ABC transporter, but also the *NorA* gene transcription. Wang et al. [143] found that silybin inhibited the MRSA efflux system by reducing the expression of *NorA* and *QacA/B*, thereby destroying MRSA resistance to antibiotics. Alves Borges Leal et al. [144] found that synthetic chalcones significantly reduced the MIC of ethylquinoline bromide and norfloxacin against drug-resistant golden grape balls by inhibiting the activity of the *NorA* efflux pump.

## 4. Antibacterial Activity of Flavonoids Combined with Antibiotics

Studies have shown that flavonoids have a synergistic effect with antibiotics, and can enhance the inhibitory effect of antibiotics on bacteria (Table 2). The fractional inhibitory concentration index (FICI) is commonly used to evaluate the combined effects of antibiotics and other drugs. When the FICI ≤ 0.5, it indicates that the combined effect of the two drugs is significantly better than their individual effects, suggesting a synergistic interaction between the drugs [145]. Yi et al. [146] found that the combination of naringenin and amikacin had a synergistic effect on *E. coli* ATCC 25922 and C7F3, which with the FICIs was 0.3125 and 0.1875, respectively. Naringenin and amikacin exert this synergistic inhibitory effect by disrupting the integrity of bacterial cell walls and membranes, leading to the leakage of cell contents. Lan et al. [141] found that chrysosplenetin, penduletin, and chrysoeriol exhibited synergistic activity when combined with norfloxacin against effluxing fluoroquinolone-resistant strain SA1199B, with FICIs of 0.375, 0.079, and 0.266, respectively. This synergistic effect is attributed to the inhibition of bacterial efflux systems by flavonoids, preventing the bacteria from expelling the antibiotics that enter their cells, thereby allowing the antibiotics to effectively kill the bacteria. Bakar et al. [147] found that flavones significantly enhanced the inhibitory effect of oxacillin on vancomycin-intermediate *S. aureus* (VISA) ATCC 700699 in a concentration-dependent manner. The FICI for the combination of flavones and nafcillin was 0.126. Cheng et al. [148] found that baicalin enhanced the damage of colistin to the bacterial cell membrane by inhibiting the activity of the bacterial efflux pump, thus playing a synergistic antibacterial role with colistin. When colistin was combined with baicalin (1024 μg/mL), mcr-1 positive *E. coli* strains recovered their sensitivity to colistin. Eumkeb et al. [56]. have demonstrated that galangin exhibits a notable synergistic effect when combined with ceftazidime. Galangin significantly inhibits the activity of penicillinase and β-lactamase, thereby reversing bacterial resistance to β-lactam antibiotics. Zhong et al. [149] discovered that 7,8-dihydroxyflavone, myricetin, and luteolin can significantly enhance the antibacterial efficacy of colistin. Further studies revealed that these flavonoids disrupt bacterial iron homeostasis by converting ferric iron to ferrous iron. The accumulation of excessive intracellular ferrous iron modulates the membrane charge of bacteria by interfering with the two-component system pmrA/pmrB, thereby promoting the binding of colistin and subsequent membrane damage.

In addition to the above flavonoids, quercetin [150,151,152,153,154], rutin [155,156], apigenin [157], luteolin [158,159,160], isoquercitrin [161], catechins [162], genistein [163], chrysin [164], EGCG [117,165,166,167], and other flavonoids can enhance the antibacterial effect of antibiotics, and the methods used in these studies are different, but FICI analysis shows that the combination of these flavonoids and antibiotics has a synergistic antibacterial effect.

Despite extensive evidence demonstrating the synergistic effects of flavonoids with antibiotics, it is important to note that flavonoids may also exhibit antagonistic interactions under certain conditions. For instance, hesperetin and naringenin, which exhibit antibacterial activity against both methicillin-sensitive *S. aureus* (MSSA) and methicillin-resistant *S. aureus* (MRSA), have been shown to have their antibacterial effects counteracted when combined with β-lactam antibiotics such as methicillin, penicillin, and oxacillin [168]. These antagonistic effects highlight the complexity of flavonoid-antibiotic interactions, which can be influenced by various factors, including bacterial species, antibiotic mechanisms of action, and flavonoid concentrations. Therefore, careful evaluation of flavonoid–antibiotic combinations is essential to avoid potential antagonism that could compromise therapeutic efficacy.

**Table 2:** Part of the synergistic effect of flavonoids and antibiotics.

		MICs (μg/mL)			
Strains	Agents	Alone	Combination	FICI	References
*S. aureus* 1199B	Chrysosplenetin	>256	32	<0.375	[141]
	Norfloxacin	32	8		
	Penduletin	>128	2	<0.079	
	Norfloxacin	32	2		
	Chrysoeriol	>128	2	<0.266	
	Norfloxacin	32	8		
*E.coli* C7F3	Naringenin	2000	250	0.1875	[146]
	Amikacin	64	4		
* S. aureus * ATCC 700699	Flavone	1600	100	0.094	[147]
	Vancomycin	8	0.25		
	Flavone	1600	100	0.126	
	Oxacillin	800	50		
* E. coli * HZ−46	Baicalin	>2048	256	0.25	[148]
	Colistin	2	0.25		
*Aeromonas hydrophila* (*A. hydrophila*)	Quercetin	360	90	0.28	[9]
	Florfenicol	2.5	0.078		
* P. aeruginosa * O1	Quercetin	500	62.5	0.25	[150]
	Tobramycin	4	0.5		
	Quercetin	500	62.5	0.25	
	Amikacin	8	1		
	Quercetin	500	125	0.375	
	Ceftriaxone	8	1		
	Quercetin	500	125	0.375	
	Gentamycin	4	0.5		
	Quercetin	500	125	0.375	
	Levofloxacin	2	0.25		
* Acinetobacter baumannii (A. baumannii) ColR-Ab4 *	Quercetin	256	16	0.1875	[152]
	Colistin	32	4		
*S. aureus* ATCC33591	Quercetin	>1024	4	0.5	[154]
	Tetracycline	32	16		
	Quercetin	>1024	4	0.25	
	Doxycycline	32	8		
MRSA	Apigenin	32.5–62.5	/	0.18–0.47	[157]
	Ampicillin	800	107		
	Apigenin	32.5–62.5	/	0.18–0.47	
	Ceftriaxone	58	2.6		
*A. hydrophila* MTCC 646	Rutin	1100	275	0.5	[155]
	Florfenicol	16	4		
* S. aureus * ATCC 25923	Luteolin	62.5	3.9	0.125	[159]
	Ampicillin	15.63	0.97		
*E.coli* DMST 20661	Luteolin	200	80	<0.47	
	Amoxicillin	>1000	70		
* A. baumannii *	Chrysin	>128	1–8	0.047–0.256	[163]
	Colistin	0.125–16	0.031–0.125		
*P. aeruginosa*, *A. baumannii*	Kaempferol	≥512	4–16	0.031–0.266	[164]
	Colistin	32–128	1–16		
*Vibrio cholerae (V. cholerae) N16961 Vibrio cholerae*	EGCG	125	0.97	0.009	[165]
	Tetracycline	3.91	0.004		
MDR *E.coli*, *S. aureus*	EGCG	625–1250	78.125–156.25	0.325	[166]
	Gentamicin	32	6.4		
Extended-Spectrum Beta-Lactamase *E.coli*	EGCG	1500	50	0.1	[167]
	Cefotaxime	128	8		

## 5. Conclusions

This review summarizes the individual antibacterial activity of flavonoids, their combined antibacterial effects with antibiotics, and their underlying antibacterial mechanisms. Among the various subclasses of flavonoids, flavones, flavonols, and flavanones encompass a greater number of compounds with good antibacterial activity compared to flavanols, anthocyanins, and isoflavones. Furthermore, when flavonoids are combined with different antibiotics, they can exert synergistic effects, enhancing the therapeutic efficacy of the antibiotics. Notably, the combinations of penduletin with norfloxacin, quercetin with colistin, and EGCG with tetracycline demonstrate particularly remarkable synergistic effects which warrant further investigation. Additionally, this review discusses the antibacterial mechanisms of flavonoids, including the disruption of bacterial cell membranes and cell walls, interference with bacterial nucleic acids and proteins, inhibition of biofilm formation and virulence factor production, modulation of bacterial signaling pathways, and suppression of bacterial efflux systems. We found that many flavonoids exert their antibacterial effects through multiple mechanisms of action. For example, quercetin, a representative flavonol, not only compromises bacterial integrity, but also inhibits bacterial enzyme functions and virulence factor production. The diversity of these mechanisms contributes to reducing the development of bacterial resistance.

These findings suggest that flavonoids may play a crucial role in the development of novel antibacterial agents and combination therapies to address the growing challenge of antibiotic resistance. However, many of these compounds have only been tested in vitro. Their antibacterial efficacy needs to be further validated in vivo to assess their effectiveness and safety in complex biological environments. Furthermore, in-depth research into the pharmacological mechanisms, toxicological profiles, and potential resistance issues of these compounds is necessary to provide a solid scientific foundation for their future clinical applications.

## Data Availability

Data sharing is not applicable to this article as no datasets were generated or analyzed during the current study.
